# Formation and Development of Pore Structure in Transitional Shale—Insights from Thermal Simulation Experiments on the Shanxi Formation Shale in the Ordos Basin

**DOI:** 10.1021/acsomega.5c01831

**Published:** 2025-05-16

**Authors:** Xuemin Xu, Xiaotao Zhang, Bin Shen, Zhichao Xu, Jiajia Yang, Jing Qin, Caizhi Hu, Peng Fang, Shizhen Li, Taotao Cao, Yanran Huang

**Affiliations:** † National Research Center for Geoanalysis, 201393 Chinese Academy of Geological Science, Beijing 100037, China; ‡ Oil and Gas Survey, China Geological Survey, Beijing 100083, China; § School of Earth Sciences and Spatial Information Engineering, Hunan University of Science and Technology, Xiangtan, Hunan 411201, China

## Abstract

Transitional shale gas reservoirs have become a new field of shale gas exploration in China. However, the formation and evolution of shale pores, particularly the organic matter (OM) nanopores, in this type of shale are still unclear. Simulation experiments with a typical transitional shale were conducted, and field emission scanning electron microscopy (FE-SEM) and low pressure CO_2_ and N_2_ adsorption (CO_2_GA/N_2_GA) were used to characterize the shale pore structure. Results show that the development of OM pores is controlled by the hydrocarbon generation process, and meanwhile, the evolution model in the transitional shale is established. At the oil generation stage (0.5% < Ro < 1.1%), volumes of micropores and mesopores decrease rapidly, with a minimum value of micropore volume at Ro = 1.02%, due to the infilling of extractable OMs. At the post-oil generation stage (1.1% < Ro < 1.5%), micropore volume increases due to the cracking of extractable OM, and mesopore volume decreases owing to partial extractable OM migrating into mesopores and the effect of compaction. At the wet gas generation stage (1.5% < Ro < 2%), volumes of shale pores slightly increase due to the release of the occupied pores. At the dry gas generation stage (Ro > 2%), volumes of micropores, mesopores, and macropores increase rapidly when Ro is below 2.99%, due to the porous solid bitumen formation and new pores generated in kerogen. When Ro is above 3.31%, micropore and macropore volumes decrease due to the graphitization of OM and strong compaction. The continuous increase in mesopores is probably due to the combination of micropores. This study can provide a scientific guide for transitional shale gas exploration and resource evaluation.

## Graphical abstract

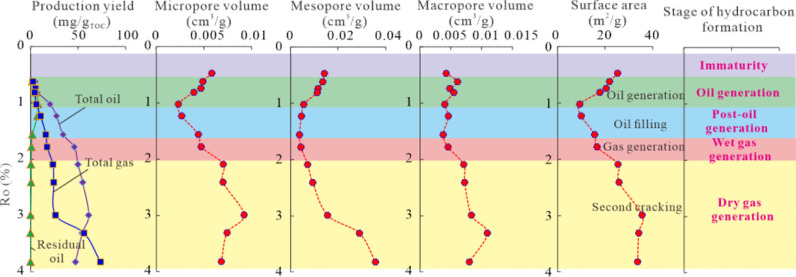

## 1 Introduction

Unconventional oil and gas, particularly shale gas, play a significant role in the energy structure and have changed the global supply structure of oil and gas to some extent.^1–3^ Increasing investment in shale gas exploration, searching for favorable shale gas exploration areas, and evaluating the potential of shale gas resources are currently urgent tasks. Organic-rich shale reservoirs have attracted many scholars with the expectation of breakthroughs in shale gas exploration across the world.^1,4–6^ However, practice has proven that not all shale layers have the conditions to form shale gas reservoirs. Taking China as an example, shale gas exploration and development have made great progress in marine shale gas reservoirs, particularly in the Wufeng–Longmaxi Formations in the Sichuan Basin,^7,8^ while no substantive breakthroughs in shale gas within transitional shale layers have yet been made. The transitional organic-rich shales are widely distributed in China, including the Ordos Basin and Bohai Bay Basin in North China, the Upper Yangtze region of the Sichuan Basin in South China, and the Junggar-Tuha and Tarim Basins in Northwest China.^8–11^ As reported, the recoverable resource of marine-continental transitional shale gas is 5.08 × 10^12^ m^3^; however, this figure is roughly agreed upon by scholars due to its highly controversial.^12^ Unlike marine shale gas reservoirs, transitional shales are interbedded with sandstones and coals, are not notably concentrated at certain horizons vertically, and are unstable and cannot be continuously distributed over a wide area horizontally.^13^ Significantly, the configuration types of transitional shale are mainly source-reservoir integration and source-reservoir symbiosis.^14^ Hence, accurately characterizing hydrocarbon gas generation and its coupling relationship with reservoir space is crucial for shale gas resource evaluation.

Unlike conventional oil and gas, retained hydrocarbon gas is stored directly in nanometer-sized pores, subsequently accumulating in the shale pore system and forming a shale gas reservoir. Therefore, it is crucial to study the pore structure and its evolution characteristics in shale.^15–18^ As summarized by scholars, shale pores are divided into micropores (pore diameter <2 nm), mesopores (pore diameter between 2 and 50 nm) and macropores (pore diameter >50 nm) according to the classification scheme of the International Union of Pure and Applied Chemistry (IUPAC).^19^ Shale pore structure and evolution processes have been widely investigated qualitatively and quantitatively using a series of techniques, e.g., low-pressure N_2_/CO_2_ adsorption (N_2_GA/CO_2_GA), scanning electron microscopy (SEM), mercury injection capillary pressure (MICP), and nuclear magnetic resonance (NMR).^20–22^ These techniques have significantly improved the understanding of shale pore characteristics, evolutionary processes, geometric topological structures, gas occurrence states, and their controlling factors in shale gas reservoirs.^23–25^ The most important discovery is that organic matter (OM) pores are the dominant pore type in marine shale gas reservoirs and directly determine shale porosity, shale gas content, and the hydraulic properties of shale layers.^26,27^ Unlike marine shale, transitional shale exhibits different types and compositions of OMs across various regions and ages, resulting in different hydrocarbon gas generation capacities, OM pore development degrees, and shale gas storage capacities. Hence, carefully investigating the influence of OM composition/type on hydrocarbon generation, OM pore evolution, and their coupling relationships is strongly essential.

The lithologies of transitional strata are quite complex and highly heterogeneous due to frequent changes in sedimentary environments.^20,27^ Transitional shales have small single-layer thickness but large cumulative total thickness, often with a high abundance of OM and good hydrocarbon gas generation potential.^28–30^ Unlike marine shales, transitional shales exhibit wider variations in lithofacies assemblages and key geological parameters, with relatively poor reservoir conditions, lower gas content, and greater variability in gas content.^14^ Transitional shales in different strata, even within the same stratum, show significant differences in TOC content, pore types, and gas content among various types of transitional shales, and the exploration experience of marine shale gas is difficult to adapt to the complex geological conditions of transitional shale.^31,32^ Previous studies have indicated that transitional shales contain diversified pores with weak connectivity, and show greater heterogeneity in three-dimensional spaces due to high clay minerals and complex OM compositions.^33,34^ Clay mineral content is often greater than 50%, which can significantly increase shale porosity. Transitional shale is primarily dominated by type II_2_–III kerogen, which is a pivotal factor in pore development.^35–37^ Many studies have reported that the primary factors controlling OM pore development are thermal maturity and maceral composition. It has been determined that OM pores in marine shales begin to generate as Ro = 0.9% and decay as Ro > 3.0–3.5%.^38,39^ However, the evolution process of type I and II_1_ kerogens is different from that of type III kerogen. The former has been well studied, and it can be concluded that the evolution process involves kerogen first depolymerizing into macromolecular-based soluble intermediates (e.g., asphaltene and resin) and then decomposing into hydrocarbons, while the latter follows a “defunctionalization” reaction mechanism.^40^ Thus, transitional shale with type III kerogen may have a different pore evolution pattern with increasing maturity compared to marine shale. Influenced by organic macerals, transitional shales generally exhibit lower organic pore development.^41^ In addition, high- and overmature transitional shales are widely distributed in China. For instance, the Ro value of Carboniferous-Permian transitional shale exceeds 4% in the Qinshui Basin and is between 4% and 5% in the western Henan area, China.^42^ However, it remains an open question whether these transitional shales have shale gas potential. Meanwhile, there is a lack of systematic research on OM pore evolution in transitional shale, and the risk maturity threshold for shale gas exploration is still unknown.

Artificial thermal simulation experiments have been widely applied to study the pore evolution of shale^43–45^ because the short-time, high temperature conditions can reproduce geological processes. In this study, to investigate the response of OM pore evolution in transitional shale and avoid the influence of shale composition heterogeneity on pore development, thermal simulation experiments, which can produce a series of samples with various maturities, were performed on immature shale from the Shanxi Formation in the Ordos Basin. OM pore morphologies and development characteristics were observed using a field emission scanning electron microscopy (FE-SEM) instrument, and pore structure characteristics were evaluated through N_2_GA/CO_2_GA tests. Moreover, the source-reservoir coupling mechanism was analyzed by investigating the relationship between pore evolution and hydrocarbon generation. The purpose of this work is to provide a scientific guide for transitional shale gas exploration and development at high to over-mature stages.

## 2 Sample and Methods

### 2.1 Sample

The shale sample used for thermal simulation experiments was collected from the Permian Shanxi Formation at the Palougou section in the Baode area, located on the northeast margin of the Ordos Basin (Figure 1). The Ordos Basin is the second-largest sedimentary basin in North China, and the Shanxi Formation is considered one of the most important layers for transitional shale gas exploration in recent years. The thickness of the Shanxi Formation is generally 80–90 m, and the lithology is mainly composed of gray-light yellow conglomeratic sandstones, middle-fine sandstone, thin carbonaceous mudstone, and black shale (Figure 1). Based on the lithological association, sedimentary cycle, and coal-bearing properties, the Shanxi Formation is divided into the Shan 2 and Shan 1 members. The PLG sample was collected from the Shan 2 member due to its low thermal maturity and high total organic carbon (TOC) content. As shown in Table 1, the PLG sample has a high TOC content of 18.56% and a low maturity value with Ro = 0.47%. The δ^13^C_org_ value of the studied sample is −24.3‰, and therefore the OM is considered to be type II_2_–III kerogen. In addition, the studied sample has good hydrocarbon generation potential, as indicated by the value of S_1_ + S_2_ (52.05 mg/g), suggesting it is very suitable for thermal simulation experiments.

**Figure 1:**
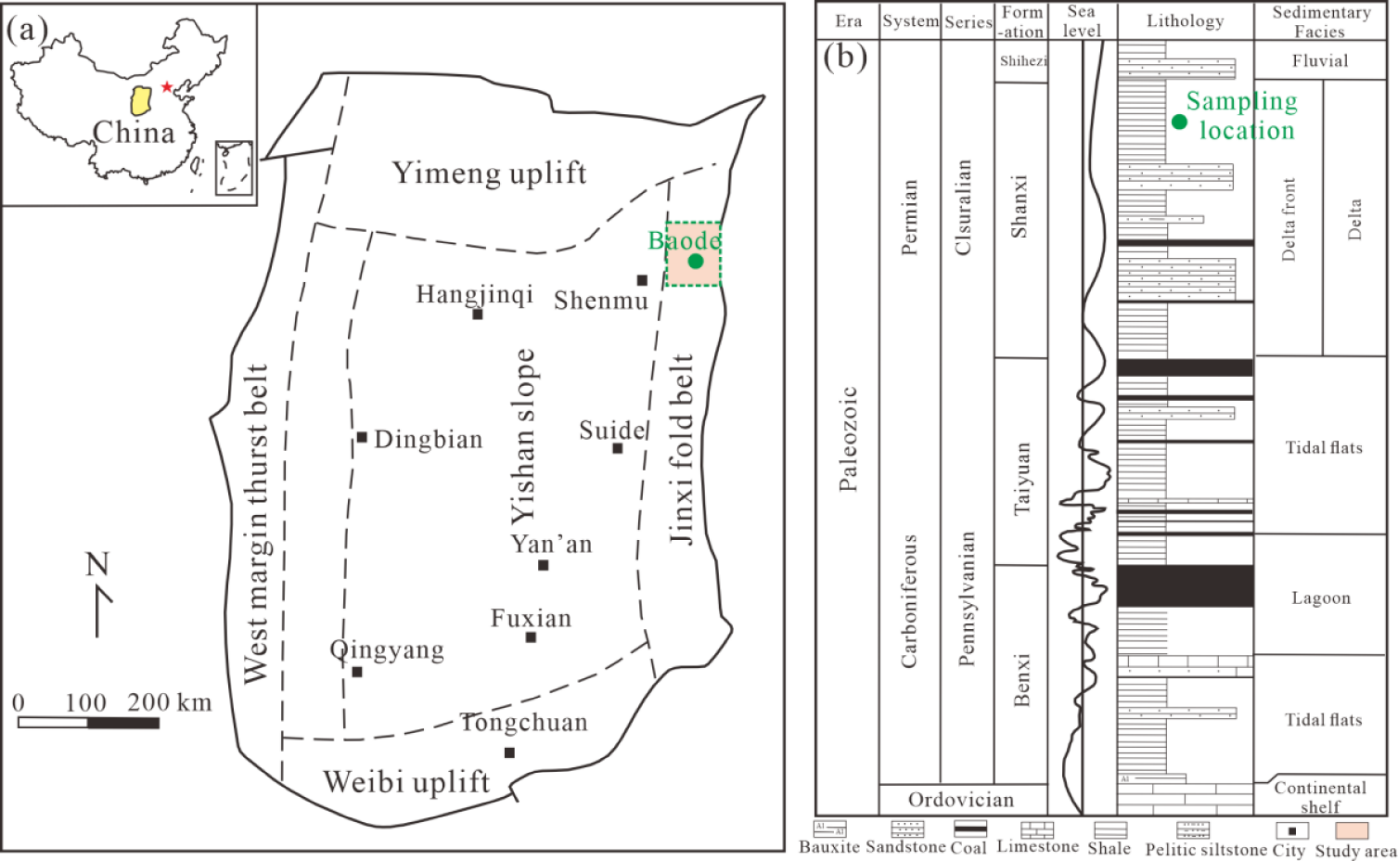
Location of the sampling area and stratigraphic column. (a) Regional tectonics of the Ordos Basin (adapted with permission from ref. 46 Copyright 2024 ACS), and (b) Carboniferous-Permian stratigraphy in terms of well L-2–48 (adapted with permission from ref. 32 Copyright 2023 Elsevier).

**Table 1:** Organic Geochemical Parameters of the PLG Sample of the Shanxi Formation

Sample	TOC (%)	S_1_ (mg/g)	S_2_ (mg/g)	S_1_ + S_2_ (mg/g)	*T*_max_ (°C)	HI (mg/g)	OI (mg/g)	δ^13^C_org_ (‰)	Ro (%)
PLG	18.56	0.40	51.65	52.05	426	281	16	–24.3	0.47

### 2.2 Methods

#### 2.2.1 Sample Preparation

The studied sample was prepared, and all the measurements were performed at the National Research Center for Geoanalysis, Beijing, China. Before the tests, the shale sample was polished with gauze, washed with deionized water, and dried in a fume hood. The prepared sample was crushed into 8–20 mesh sizes to ensure complete homogenization and was finally divided into 12 portions, with each portion weighing 120–150 g. Each sample underwent a thermal simulation experiment according to the established method. First, the pressure and temperature of the experiments were set based on the burial depth and thermal evolution history of the Shanxi Formation in the central depositional area of the Ordos Basin. Second, the thermal simulation experiments were conducted, and the oil and gas were collected using the episodic hydrocarbon collection method. Finally, the simulated samples were used for organic geochemical measurements, FE-SEM observations, and N_2_/CO_2_ adsorption tests.

#### 2.2.2 Thermal Simulation Experiment and Product Collection

To better understand the evolution of hydrocarbon generation and expulsion, variations in geochemical parameters, and the evolution process of OM pores, a series of thermal simulation experiments were conducted using a semiclosed system with a thermocompression hydrocarbon generation and expulsion simulator. The detailed experimental procedure can be referred to in previous studies.^44^ The pressures and temperatures of the experiments are given in Table 2. Accordingly, 12 temperature points selected for the thermal simulation experiments are 275 °C, 300 °C, 320 °C, 340 °C, 355 °C, 385 °C, 400 °C, 425 °C, 450 °C, 500 °C, 550 °C, and 600 °C, with a heating rate of 2 °C/min. During the experiments, each temperature setting was maintained for 72 h, after which the sample cell was allowed to cool naturally, and oil and gas hydrocarbons were collected once the sample cell had cooled completely . The values of strata and lithostatic pressures were also determined based on the burial and thermal history data of the Shanxi Formation in the depositional center of the Ordos Basin to ensure that the results of the simulated experiments closely approximate real geological conditions. It should be noted that the expelled oil includes oil collected from the liquid collector and the eluted oil washed from the sample surface using dichloromethane. The residual oil was measured by extracting the soluble OM from the simulated samples by using a Soxhlet instrument.

**Table 2:** Simulation of Temperature-Pressure Conditions for the Simulated Experiments

Sample ID	Temperature (°C)	Strata pressure (MPa)	Lithostatic pressure (MPa)	Heating rate (°C/min)
PLG-275	275	28	67.2	2
PLG-300	300	29	70	2
PLG-320	320	30	72	2
PLG-340	340	32	76.8	2
PLG-355	355	35	84	2
PLG-385	385	53.7	85.92	2
PLG-400	400	54.6	87.36	2
PLG-425	425	55.5	88.8	2
PLG-450	450	57.6	92.16	2
PLG-500	500	59.85	95.76	2
PLG-550	550	61.95	99.12	2
PLG-600	600	64.05	102.48	2

#### 2.2.3 Rock-Eval Pyrolysis, TOC Test, and Vitrinite Reflectance Analysis

The pyrolysis test was conducted using a Rock-Eval 6 apparatus (Vinci Technologies, France) in accordance with the industry standards of GB/T18602-2012, and the measurement procedure can be referred to in Behar et al.^47^ A sample of approximately 100 mg was crushed to 100–200 μm and then heated to 600 °C using nitrogen as the carrier gas in the apparatus to obtain organic geochemical parameters, i.e., maximum cracking temperature (*T*_max_), free hydrocarbon (S_1_) cracking hydrocarbon (S_2_), hydrogen index (HI), and oxygen index (OI).

Original and simulated samples were crushed to 150 μm and subsequently analyzed using a LECO CS-344 instrument (LECO, USA) to determine the TOC content in accordance with the Chinese national standard GB/T 19145-2022. Approximately 100 mg of powdered sample was weighed and decarbonized by HCl to remove inorganic carbonate. The residue was washed with DI-H_2_O and dried at 60 °C overnight. After this treatment, the samples were measured with the CS-744 instrument. During the experimental process, CO_2_ was released as a result of the combustion of OM at approximately 1100 °C, and this was used to calculate the TOC content based on the amount of CO_2_ produced.

Samples were prepared as polished pellets for vitrinite reflectance (Ro) measurements using a Zeiss Axioimager II microscope system equipped with an ultraviolet light source and a Diskus-Fossil system. Ro values were determined by using an ultrafine pixel size (0.1 μm) probe under oil immersion conditions in accordance with the Chinese national standard SY/T 5124-2012.

#### 2.2.4 Gas Product Analysis

Hydrocarbon gas and nonhydrocarbon gas were gathered according to the predesigned scheme and then measured by the Agilent 7890B GC instrument equipped with a flame ionization detector (FID) and two thermal conductivity detectors (TCDs). The test was performed in accordance with the Chinese national standard GB/T 13610-2020. During the experiment, the oven temperature for the gas test was initially held at 60 °C for 1 min, increased first to 80 °C at a rate of 20 °C/min, subsequently increased to 190 °C at a rate of 30 °C/min, and then held at 190 °C for 2 min. The volume of gas products was quantified by three laboratory standard gas samples, with a deviation of 0.1%.

#### 2.2.5 N_2_GA and CO_2_GA Measurements

N_2_GA measurement on shale samples was conducted using a Micromeritics ASAP 2460 surface area and pore analyzer to obtain primarily mesopore and macropore distribution, volume, and surface area. Prior to the test, the samples were crushed to 100 μm and subsequently dried at 110 °C for 12 h under vacuum to remove free water and volatile gases. The treated samples were analyzed using supercritical nitrogen gas (−196 °C) and a series of precisely controlled gas pressures. The Brunauer-Emmett-Teller (BET) method and the Barrett-Joyner-Halenda (BJH) method were used to obtain the surface area and volume of shale pores.^48,49^

CO_2_GA measurement was performed to obtain information on micropores (0.3–1.48 nm) using a NOVA4200e specific surface area and pore size distribution analyzer. Samples were ground to a size of 100 μm and then dried to degas and dewater for approximately 12 h at 110 °C. The adsorption and desorption data of CO_2_ were collected at different relative pressures (0.0001–0.03) at a temperature of 273.15 K. The Dubinin-Radushkevich (D-R) method was adopted to calculate the surface area of micropores, and the Dubinin-Astakhov (D-A) method was used to obtain information on pore volume and pore size distribution of micropores.

#### 2.2.6 FE-SEM Observation

A visual depiction of pore distribution, morphology, size, and location can be observed by FE-SEM observations. Original and simulated samples were first polished by ion milling to obtain smooth surfaces and were subsequently coated with a layer of carbon with a purity of 99.99% and a thickness of 5 nm. The treated samples were measured by a ZEISS GEMINI 2 FE-SEM instrument at a temperature of 24 °C and a relative humidity of 35%, equipped with Energy Dispersive Spectroscopy (EDS), which was used for elemental analysis.

## 3 Results

### 3.1 Organic Geochemical Variations during the Thermal Simulation Process

Organic geochemical features of all the simulated samples, including Ro, TOC content, and Rock-Eval pyrolysis parameters, are listed in Table 3. The Ro value of the simulated samples varies from 0.62% to 3.82% (Figures 2 and 3), which exhibits a significant linear correlation with simulated temperature points. The result differs from previous data on the Yan’an Formation shale with a similar kerogen type conducted in a closed system.^50^ The Ro value of simulated samples from the Shanxi Formation shows a rapid increase with increased simulated temperature, with a correlation coefficient R^2^ = 0.9911. However, that from the Yan’an Formation increases slowly, probably due to the closed system, with a correlation coefficient R^2^ = 0.7597, reflecting that the closed system can inhibit the increase of thermal maturity. This suggests that hydrocarbon generation in actual geological conditions is semiopen, and hence, a relatively accurate Ro value can be obtained in this study using a semi-open thermal simulation system.

**Table 3:** Organic Geochemical Parameters of Simulated Samples under Different Pyrolysis Temperatures

Sample ID	Ro (%)	TOC (%)	S_1_ (mg/g)	S_2_ (mg/g)	S_1_ + S_2_ (mg/g)	*T*_max_ (°C)	HI (mg/g)
PLG-275	0.62	15.99	0.49	30.34	30.83	427	190
PLG-300	0.74	17.64	1.51	48.28	49.79	437	274
PLG-320	0.81	16.49	1.97	41.3	43.27	438	275
PLG-340	1.02	17.8	5.87	35.14	41.01	444	265
PLG-355	1.23	17.34	4.66	30.59	35.25	447	205
PLG-385	1.56	14.15	1.91	12.58	14.49	508	89
PLG-400	1.78	14.57	1.34	8.22	9.56	549	56
PLG-425	2.09	14.54	1.06	3.92	4.98	578	27
PLG-450	2.41	12.97	0.81	1.99	2.8	598	15
PLG-500	2.99	11.84	0.47	0.43	0.9	607	4
PLG-550	3.31	10.81	0.64	0.27	0.91	/	2
PLG-600	3.82	10.76	0.29	0.13	0.42	/	1

**Figure 2:**
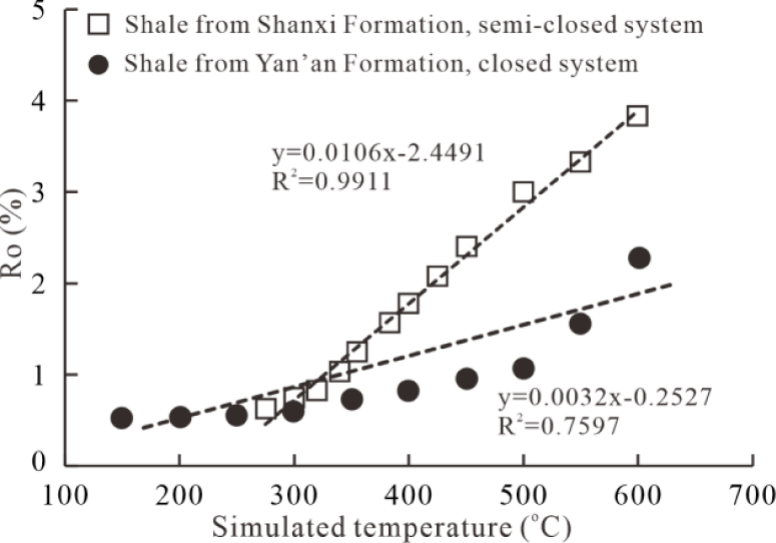
Variations of Ro values with increased simulated temperature. The data in this study are from simulated samples of the Shanxi Formation, and the comparative data are collected from simulated samples of the Yan’an Formation.

**Figure 3:**
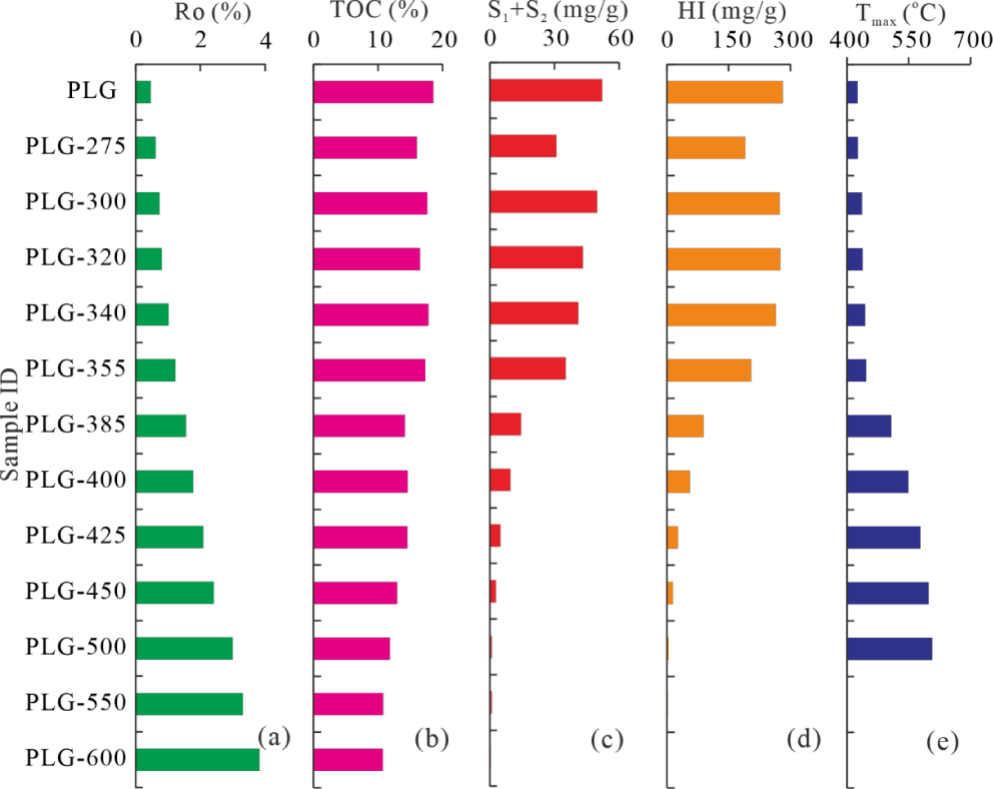
Organic geochemical features of the original sample and simulated samples at different simulation temperatures. (a) Ro; (b) TOC; (c) S_1_ + S_2;_ (d) HI; (e) *T*_max_.

As the temperature increases, the TOC content of the samples shows a gradually decreasing trend, decreasing from 18.56% for the original sample PLG to 10.76% for the sample PLG-600 at a temperature of 600 °C (Figure 3). Overall, the significant decrease in TOC content during the thermal simulation experiments occurs mainly at an Ro value >2.09%, probably due to the abundant conversion of OM into gas at this stage. This also indicates that type III kerogen primarily generates hydrocarbon gas, which results in a rapid decrease in TOC content when the simulation temperature is higher than 425 °C, corresponding to the overmature stage.

S_1_ + S_2_ and HI values show significant variations with increased simulation temperature (Figure 3). The value of S_1_ + S_2_ exhibits a drastic decrease with the increase in temperature (Figure 3c). It first shows a slight decreasing trend when the simulation temperature increases to 300 °C, followed by a slow decreasing trend as the simulated temperature rises from 300 to 355 °C, and then a significant decreasing trend when the simulated temperature exceeds 355 °C. The S_1_ + S_2_ value approaches zero when the simulation temperature increases to 500 °C. Variation in the HI value is strongly consistent with S_1_ + S_2_ value, suggesting that liquid hydrocarbons are mostly generated before the simulation temperature reaches 355 °C (Figure 3d).

The *T*_max_ value is another parameter that characterizes the thermal maturation level when the source rock is at the immature to low-mature stage. As shown in Figure 3e, the *T*_max_ value shows a slight increase when the simulation temperature rises from 275 to 355 °C and then increases rapidly to an extremely high value at temperatures of 385–500 °C. However, no data can be obtained when temperatures rise to 550–600 °C, reflecting that the *T*_max_ value is invalid when the source rock enters overmature stages.

The *T*_max_ value and HI value of the simulated samples are plotted in Figure 4. The OM type of the original sample is considered to be type II_2_ kerogen, which is consistent with the results of the carbon isotope analysis of OM. However, with increased temperature, the *T*_max_ value increases significantly, and the HI value decreases drastically; therefore , the OM is gradually converted into type III kerogen due to the aromatization of OM.

**Figure 4:**
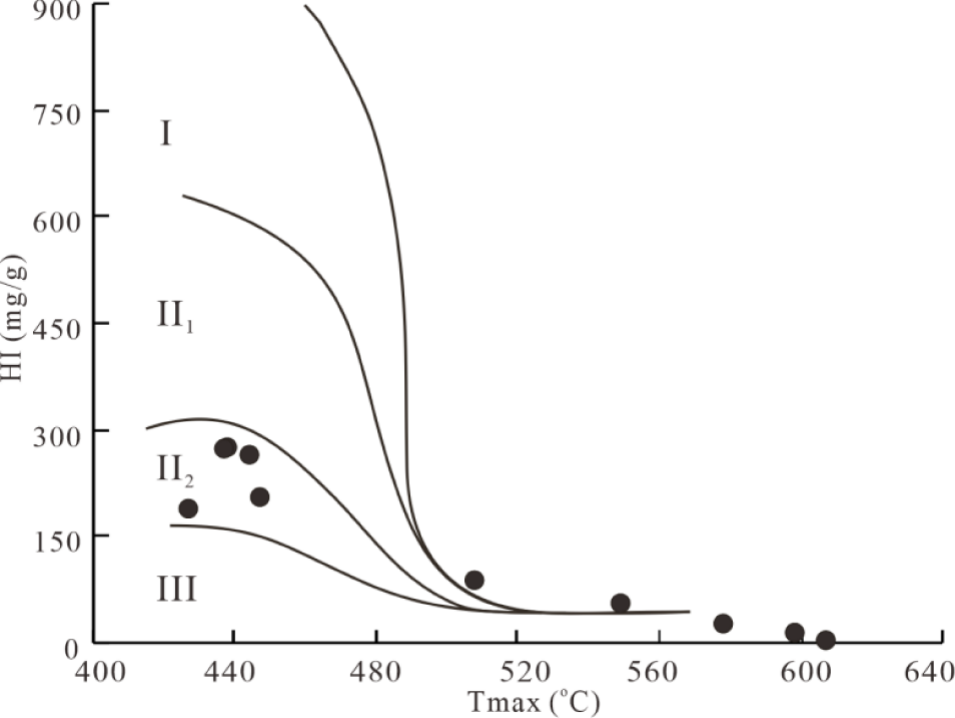
Correlation of *T*_max_ and HI indicates the evolution of the kerogen type for the simulated samples.

### 3.2 Oil and Gas Yields during the Simulation Experiments

Total oil yield, expelled oil yield, and residual oil yield during the simulation experiments are presented in Table 4 and Figure 5. With the increase in simulation temperature, the total oil yield increases slowly at temperatures ranging from 275–320 °C. As the simulation temperature increases to 340–400 °C, the total oil yield exhibits a significant increase, rising from 20.34 mg/g_TOC_ to 45.92 mg/g_TOC_. Subsequently, the yield of total oil slows down , with values changing from 49.73 mg/g_TOC_ to 60.95 mg/g_TOC_ at temperatures of 425–500 °C. However, the yield of total oil decreases from 60.95 mg/g_TOC_ to 47.15 mg/g_TOC_ when the temperature continues to increase to 600 °C. Unlike total oil yield, residual oil mainly exists in the simulated sample only at temperatures below 400 °C. Residual oil yield displays an increasing trend up to 340 °C, reaching its peak value at 340 °C, and subsequently decreases rapidly to approximately zero at 425 °C. Comparably, the variation and values in expelled oil yield are highly consistent with total oil yield, suggesting that most of the generated oil had been expelled.

**Figure 5:**
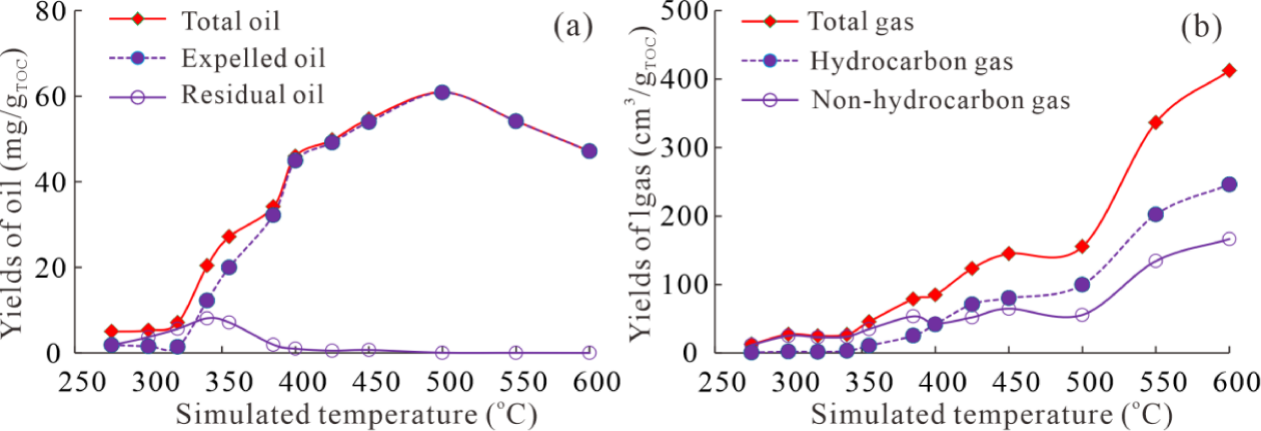
Variations of the oil yield (a) and gas yield (b) with an increased simulation temperature.

**Table 4:** Oil and Gas Production Characteristics of the Shanxi Formation Shale at Different Simulation Temperatures

Sample ID	Ro (%)	Total oil (mg/g_TOC_)	Residual oil (mg/g_TOC_)	Total gas (cm^3^/g_TOC_)	Hydrocarbon gas (cm^3^/g_TOC_)	Nonhydrocarbon gas (cm^3^/g_TOC_)
PLG-275	0.62	3.66	1.81	12.69	0.78	11.91
PLG-300	0.74	5.28	3.72	27.15	2.05	25.1
PLG-320	0.81	7.08	5.65	25.06	1.87	23.19
PLG-340	1.02	20.34	8.1	27.06	3.1	23.96
PLG-355	1.23	27.14	7.14	45.66	10.74	34.92
PLG-385	1.56	34.15	1.94	78.89	25.38	53.51
PLG-400	1.78	45.92	1.01	84.97	41.92	43.05
PLG-425	2.09	49.73	0.55	123.32	71.28	52.04
PLG-450	2.41	54.66	0.71	145.01	80.25	64.76
PLG-500	2.99	60.95	0.06	155.29	99.61	55.68
PLG-550	3.31	54.19	0.04	336.5	202.24	134.26
PLG-600	3.82	47.15	0.01	412.5	246.08	166.42

The characteristics of total gas yield, hydrocarbon yield, and nonhydrocarbon gas yield are displayed in Table 4 and Figure 5. The yields of total gas, hydrocarbon gas, and nonhydrocarbon gas show two-stage trends. First, the yields of gas increase slowly as the simulation temperature rises from 275 to 500 °C, with total gas, hydrocarbon gas, and nonhydrocarbon gas varying from 12.69 to 155.29 cm^3^/g_TOC_, 0.78 to 99.61 cm^3^/g_TOC_, and 11.91 to 55.68 cm^3^/g_TOC_, respectively, during this process. After 500 °C, the yields of total gas, hydrocarbon gas, and nonhydrocarbon gas increase rapidly from 155.29 to 412.50 cm^3^/g_TOC_, 99.61 to 246.08 cm^3^/g_TOC_, and 55.68 to 166.42 cm^3^/g_TOC_, respectively. This indicates that transitional shale has greater potential for hydrocarbon gas when it enters the overmature stage, suggesting that it is favorable for shale gas formation at the overmature stage.

### 3.3 OM Pore Variations during Simulation Experiments

Many studies have proven that the OM pore development characteristics of transitional shales are different from those of marine shales. The OM pore development degree and connectivity in transitional shale are obviously weaker than those in marine shale. OM pores in marine shale reservoirs are generally condensed into honeycomb and spongy shapes within the interior OM grains.^40,51,52^ Transitional shale, dominated by type II_2_–III kerogen, generally has a low hydrogen index and low oil generation potential, and consequently, it develops only a few OM pores with increased thermal maturation levels. For the studied original sample with an Ro value of 0.47%, OM grains are mainly derived from higher plants with dense and lumpy features, and there are many large nonporous particles in the shale matrix (Figure 6–c). Some unique OM pores, including pyrolysis pores and biostructural pores, can be observed in the FE-SEM images (Figure 6–f). Moreover, several marginal cracks related to OM grains can be observed at the edges between minerals and OMs (Figure 6). Overall, OM pores are poorly developed in the original sample PLG at the immature stage.

**Figure 6:**
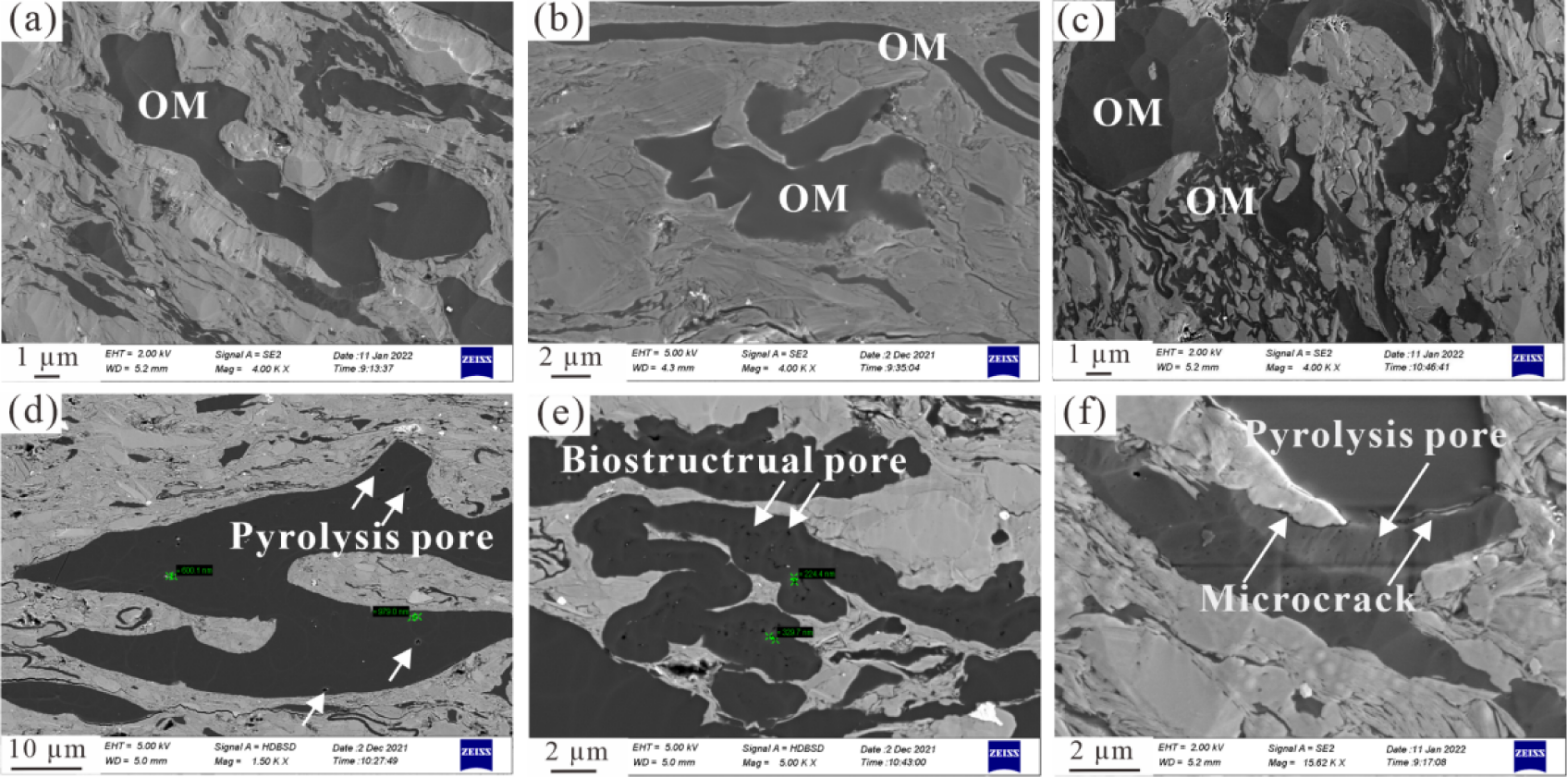
OM grains distributed within the shale matrix and OM pore development characteristics for the original sample PLG. (a)–(c) OM morphologies and distribution in the shale matrix; (d) pyrolysis pore within the OM grain; (e) biostructural pore developed within the OM grain; (f) pyrolysis pore within the OM grain and microcrack developed along the OM grain.

The development of OM pores is a function of thermal maturation.^13,14^ For marine shale reservoirs, OM pores are mainly developed within two opportune periods with Ro values of 1.11–1.53% and 2.5–2.9%, which correspond to the late oil generation stage and the cracking peak of extractable OM to gas stage, respectively.^39^ Extractable OM primarily fills OM pores and further decreases the number of OM pores during the oil generation stage and the wet gas generation stage. However, OM pore evolution characteristics during the entire hydrocarbon generation process in transitional shale are still less studied, and the effect of thermal maturation on the development of OM pores remains unclear. To investigate the variations in OM pore development with thermal maturation, seven simulated samples were selected for FE-SEM observation, which cannot fully represent OM pore development characteristics due to the heterogeneity of OM distribution. As seen in Figure 7, the degree of OM pore development is displayed with increased thermal maturation levels. At 275 °C, OM pores are not developed due to low maturity, which is similar to those of the original sample (Figure 7,b), and the influence of thermal maturity is limited at this stage. When the temperature increases to 300 °C, pyrolysis OM pores with round and irregular shapes are developed (Figure 7,d), suggesting that secondary OM pores begin to develop due to hydrocarbon generation and expulsion from OM. OM pores are rarely developed at temperatures of 320–385 °C overall (Figure 7,e–i), probably because pregenerated oil fills the OM pores, rendering them invisible under FE-SEM observation. Certainly, there are also some OM grains containing pores with smaller sizes, and even individual OM particles (probably solid bitumen) contain abundant nanometer-sized pores (Figure 7). When the temperature increases to 425 and 500 °C, secondary OM pores with larger diameters are better developed within solid bitumen particles and partial vitrinite grains (Figure 7,k–l). Therefore, newly formed OM pores result in a large increase in pore volume for transitional shale at the overmature stage, which is consistent with marine shale. Differently, the OM pores in marine shale are more developed than those in transitional shale, and the high content of vitrinite and inertinite may seriously restrict OM pore development in transitional shales.^53^

**Figure 7:**
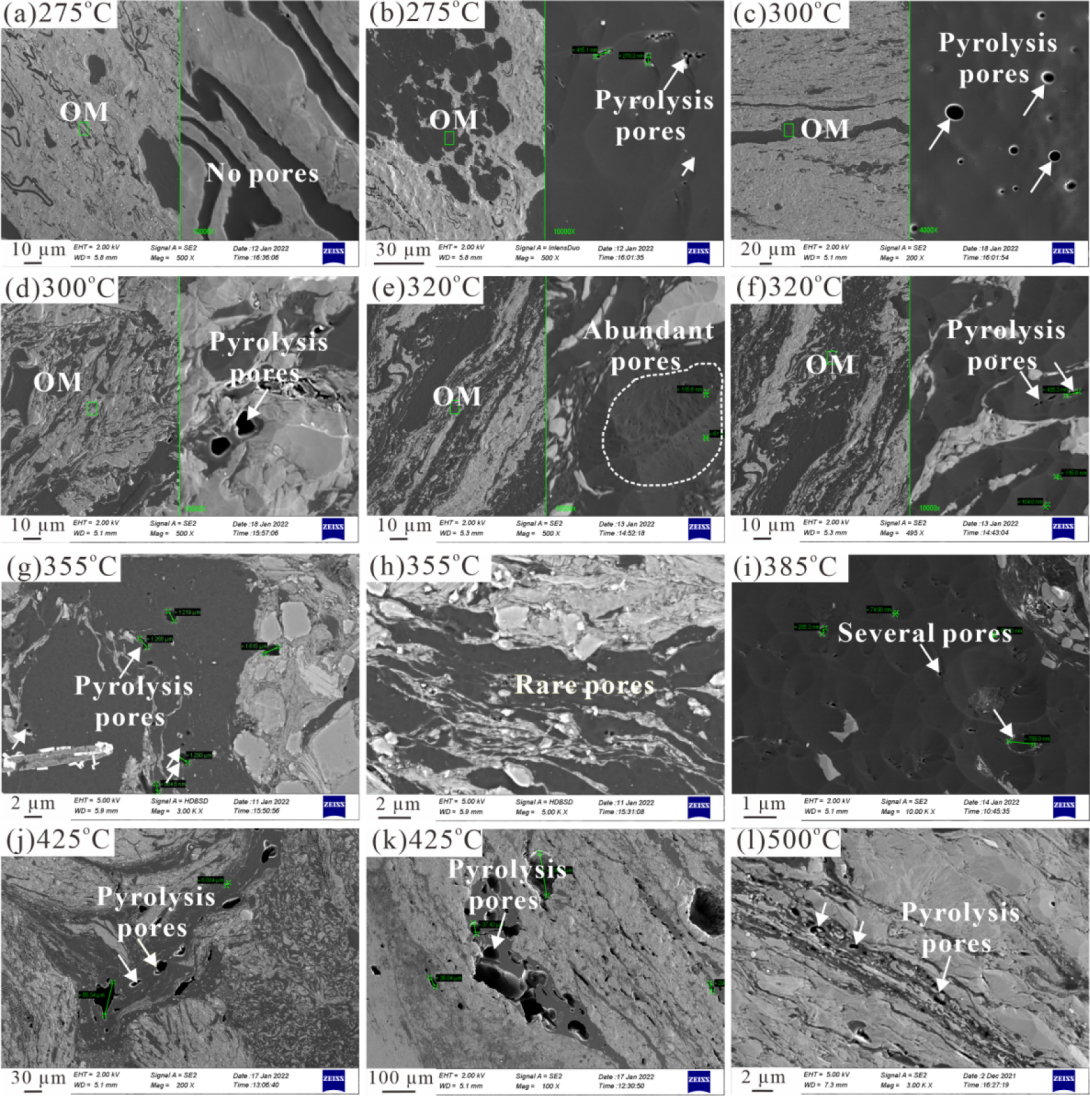
FE-SEM images showing OM pore development in simulated samples at different temperatures. (a, b) 275 °C; (c, d) 300 °C; (e, f) 320 °C; (g, h) 355 °C; (i) 385 °C; (j, k) 425 °C; (l) 500 °C.

### 3.4 Pore Size Distributions

The N_2_ and CO_2_ adsorption isotherms of the original and simulated samples are plotted in Figure 8. The CO_2_ adsorption capacity of the samples first decreases and then increases with increasing temperatures (Figure 8). The strongest adsorption capacity for CO_2_ is observed in sample PGL-500, while the weakest adsorption capacity is observed in sample PGL-340. Compared with the natural sample, the simulated samples exhibit a larger CO_2_ adsorption capacity.^54^ The largest adsorption capacity for N_2_ is observed in sample PGL-600, while the least adsorption capacity is observed in sample PGL-385. The N_2_ adsorption isotherms of the studied samples belong to type IV of the IUPAC classification and are characterized by an obvious hysteresis loop in the desorption isotherms, which is used to reflect the pore shape. The shape of the hysteresis loop is close to type H2, representing the slit-shaped pores created by the accumulation of plate-like particles in the samples (Figure 8). The pore shape remains unchanged during the entire thermal simulation experiments, suggesting that the shapes of shale pores are less influenced by the thermal evolution of OM.

**Figure 8:**
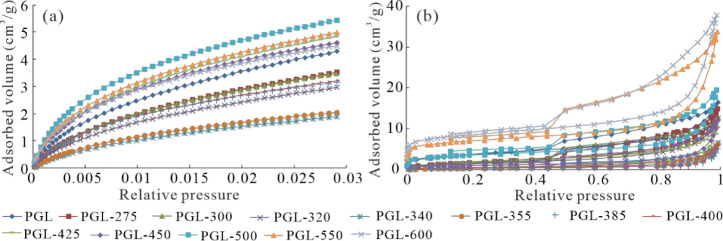
Low-pressure CO_2_ adsorption isotherms (a) and N_2_ adsorption–desorption isotherms of the original and simulated samples (b).

The volume distributions of micropores present a triple feature characterized by CO_2_ adsorption isotherms, and micropores are mainly contributed by the pores of 0.48–0.62 nm, 0.85–0.89 nm, and 0.97–1 nm (Figure 9), with the former being dominant . Mesopore and macropore volume distributions are characterized by the adsorption isotherms of N_2_GA. The development of mesopores and macropores is better than that of micropores, with mesopores being the dominant pore type (Figure 9). Distributions of the surface area of micropores, determined by the CO_2_GA test, are similar to those of pore volume (Figure 9), and the surface area characterized by the N_2_GA test is mainly contributed by mesopores with diameters of 1.9–7.1 nm and 15.11–40.67 nm (Figure 9).

**Figure 9:**
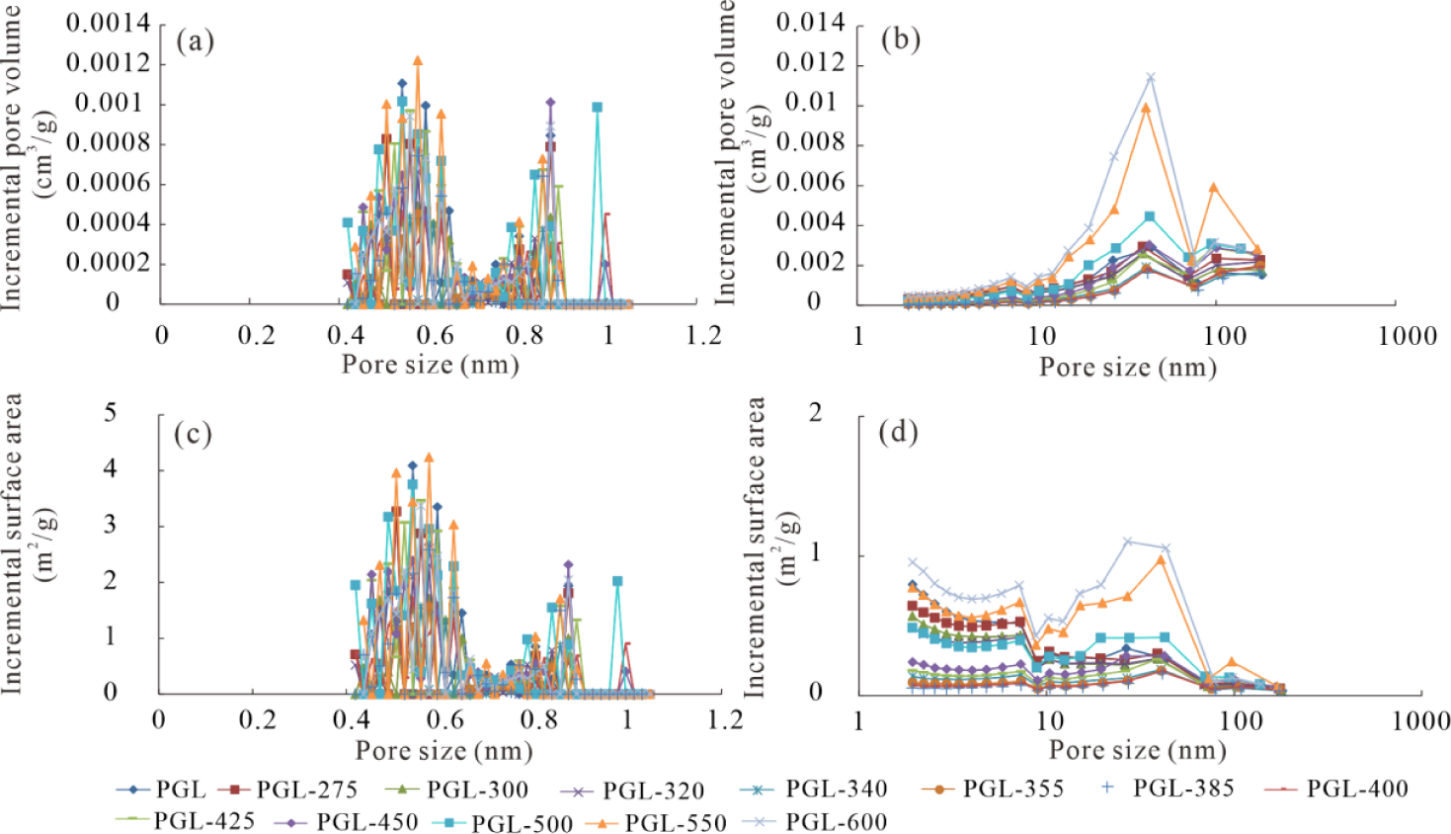
Distributions of pore volume (a-b) and surface area (c-d) of the original and simulated samples. (a) Pore volume distribution measured by CO_2_GA; (b) pore volume distribution measured by N_2_GA; (c) surface area distribution measured by CO_2_GA; (d) surface area distribution measured by N_2_GA.

### 3.5 Pore Structure Parameters

Pore volume and surface area data of the original and simulated samples are given in Table 5. CO_2_GA technique can only obtain micropore parameters, while the N_2_GA technique can also measure pores ranging from micropores with diameters larger than 1.2 nm to macropores, which cannot cover micropores smaller than 1.2 nm. Therefore, the volume and surface area of micropores are calculated jointly by CO_2_GA and N_2_GA methods, while those of mesopores and macropores are obtained from N_2_GA data. Total pore volume and surface area are the sum of the volume and surface area of micropores, mesopores, and macropores. The total pore volume and total surface area of the simulated samples vary from 0.0118 to 0.0504 cm^3^/g and from 9.29 to 35.47 m^2^/g, respectively. Different types of pores contribute differently to shale pore volume and surface area. Volumes of micropore, mesopore, and macropore vary from 0.230 to 0.0919 cm^3^/g, 0.00372 to 0.0356 cm^3^/g, and 0.0376 to 0.0109 cm^3^/g, respectively. Surface areas of micropore, mesopore, and macropore vary from 7.35 to 29.81 m^2^/g, 0.98 to 11.26 m^2^/g, and 0.13 to 0.43 m^2^/g, respectively. Therefore, mesopores play a significant role in pore volume, while micropores and macropores also contribute to the total pore volume (Figure 10). In contrast, the total surface area is mainly contributed by micropores, followed by mesopores, with macropores having a limited contribution to the total surface area (Figure 10).

**Figure 10:**
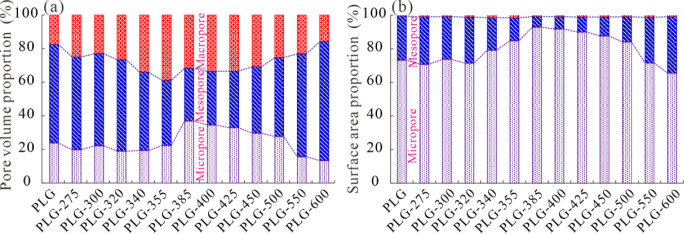
Proportions of pore volume and surface area for the original and simulated samples. (a) Pore volume proportions of micropores, mesopores, and macropores; (b) surface area proportions of micropores, mesopores, and macropores.

**Table 5:** Pore Structure Parameters of the Shanxi Formation Shale under Different Pyrolysis Temperatures

			Pore volume (cm^3^/g)			Surface area (m^2^/g)		
Sample ID	Total pore volume (cm^3^/g)	Total surface area (m^2^/g)	Micropore	Mesopore	Macropore	Micropore	Mesopore	Macropore
PLG	0.0243	25.19	0.00581	0.0142	0.00424	18.44	6.60	0.15
PLG-275	0.0245	21.81	0.00487	0.0135	0.00608	15.42	6.16	0.23
PLG-300	0.0210	20.41	0.00467	0.0115	0.00481	15.04	5.19	0.18
PLG-320	0.0206	17.75	0.00393	0.0112	0.00548	12.68	4.86	0.21
PLG-340	0.0118	9.29	0.00230	0.00551	0.00401	7.35	1.80	0.14
PLG-355	0.0118	9.94	0.00265	0.00458	0.00459	8.43	1.35	0.16
PLG-385	0.0119	15.55	0.00440	0.00372	0.00376	14.44	0.98	0.13
PLG-400	0.0136	16.57	0.00470	0.00435	0.00454	15.2	1.21	0.16
PLG-425	0.0212	25.34	0.00701	0.00716	0.00707	22.78	2.28	0.28
PLG-450	0.0235	25.78	0.00698	0.00927	0.00722	22.58	2.93	0.27
PLG-500	0.0330	35.47	0.00919	0.0155	0.00833	29.81	5.31	0.35
PLG-550	0.0472	34.04	0.00741	0.0289	0.0109	24.35	9.26	0.43
PLG-600	0.0504	33.56	0.00680	0.0356	0.0080	21.99	11.26	0.31

## 4 Discussion

It has been proven that the main factors controlling shale pore development and evolution are hydrocarbon generation due to variations in thermal maturation and mineral transformation.^17,55^ Moreover, compaction can also affect the evolution of shale pores during the entire hydrocarbon generation process.^56,57^ The following is a detailed discussion of the main factors that may affect OM pore evolution during the thermal simulation process.

### 4.1 Response of OM Pore Evolution to Hydrocarbon Generation

For a shale gas reservoir, the first consideration is how large volumes of gas can be generated, and the second is how much gas can be stored. Hydrocarbon generation from OM in shale is a complex chemical process, and three distinct stages of hydrocarbon generation within transitional shale can be summarized: (1) the decomposition of kerogen into gas and bitumen; (2) the decomposition of bitumen into oil and gas; and (3) the decomposition of oil into gas and a carbon-rich coke or pyrobitumen residue.^58,59^ The hydrocarbon generation process can promote OM pore development, and circular or bubble-like OM pores may develop as hydrocarbons are generated and expelled from OM (Figure 7). Therefore, the correlation between hydrocarbon production and pore structure is meaningful for the source-storage relationship of shale gas.

In order to deeply explore the influence of hydrocarbon generation on shale porosity, cross-plots of hydrocarbon production with pore volume were analyzed. As shown in Figure 11, volumes of micropores, mesopores, and macropores exhibit initially decreasing trends and subsequently increasing trends with increased yields of total oil. The lowest value of micropore volume occurs at a Ro value of 1.02–1.23%, and those of mesopore volume and macropore volume occur at a Ro value of 1.02–1.56% (Figure 11,c,e). This suggests that shale enters the “oil window” and generates a large amount of liquid hydrocarbon due to kerogen pyrolysis. Meanwhile, it fills OM pores and interparticle pores, significantly reducing pore volumes. Micropores have large surface areas and are easily filled by oil first; subsequently mesopores and macropores are filled as more oil is produced with increasing thermal maturation.^60,61^ The later increase in volumes of micropores, mesopores, and macropores is due to the cracking of oil and the release of hydrocarbons,^57,61^ which form nearly circular or bubble-like pores in the OM grains.

**Figure 11:**
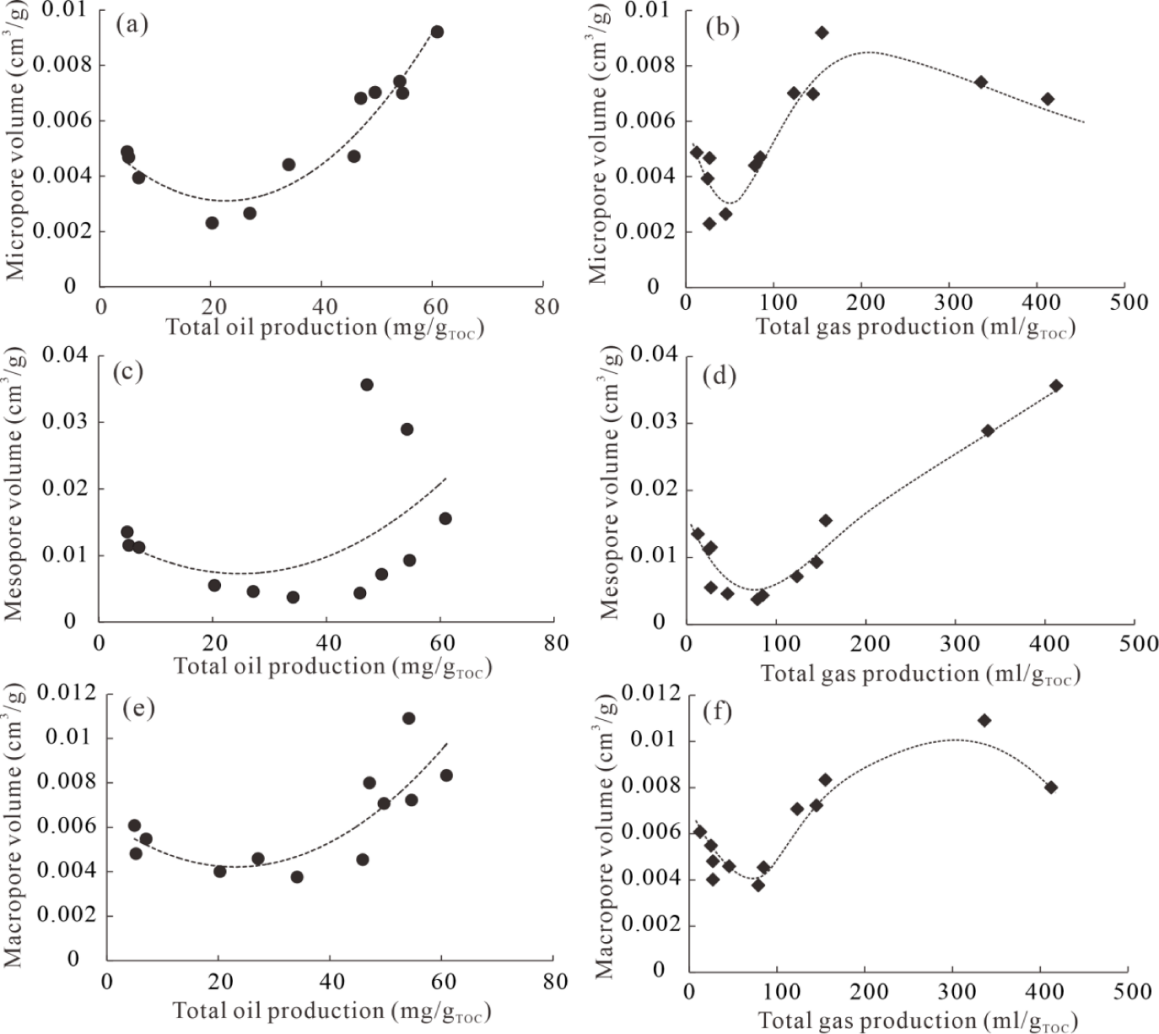
Crossplots of total oil production and total gas production with volumes of micropores (a-b), mesopores (c-d), and macropores (e-f).

Gas generation occurs throughout the entire process of thermal simulation experiments for transitional shale, including kerogen pyrolysis and oil cracking, and secondary OM pores are continuously formed before OM graphitization.^62^ As shown in Figure 11, first, micropore volume displays a rapid decrease with total gas production, reaching its lowest value at Ro = 1.02%, due to the infilling of oil. Second, with the increase in total gas production, micropore volume shows a rapid increase, likely due to the formation of solid bitumen, which is generated via kerogen and oil and creates a significant number of micropores during this process.^38^ Lastly, the slight decrease in micropore volume with increased total gas production at Ro = 3.31–3.82% is related to the transformation of micropores into mesopores. Unlike micropore volume, variations in mesopore volume can be divided into two stages, as shown in Figure 11. In the first stage, mesopore volume shows a rapid decrease due to the infilling of oil. However, an increasing trend in mesopore volume with increased total gas generation can be attributed to: (1) the generation of gaseous hydrocarbons originating from kerogen and oil, which can generate high pressures,^58,59^ thereby resisting further compaction and enhancing the growth of mesopores, and (2) the combination of micropores into mesopores with increased thermal maturation.^63^ The variation in macropore volume with increased total gas production is similar to that of mesopores before Ro = 3.31% (Figure 11), and the development mechanism of macropores is consistent with that of mesopores. However, macropore volume shows a decrease at Ro = 3.82%, likely due to the graphitization of OM and strong compaction. Jiao et al.^64^ pointed out that OM begins graphitization when Ro > 3.7%, and pore structure destruction occurs at Ro values of 3.7–4.5%. Moreover, macropores are more prone to collapse due to postcompaction during the burial process.^65^

### 4.2 Response of OM Pore Evolution to Extractable OM

Extractable OM often constitutes a significant portion of OM in organic-rich shale with a low maturation level, and hence it can have an obvious influence on shale pore evolution. Extractable OM appears as liquid hydrocarbon, free oil, or free hydrocarbon that is retained within shales. A large amount of extractable OM is generated during the oil generation and wet gas stages.^39^ By extracting shale samples, the pore volume shows a significant increase.^66^ Wu et al.^54^ revealed that liquid hydrocarbon exists in thermally simulated samples in veinlet and droplet shapes at simulation temperatures of 350–500 °C. Li et al.^67^ indicated that extractable OM exhibits different roles in different pores, with smaller pores being more easily filled and occupied by extractable OM. Additionally, some OM pores filled by extractable OM can reappear after the extractable OM is removed. Moreover, the influence of extractable OM on pore structure is also controlled by the thermal maturation stage. The impact of extractable OM on shale porosity is strongest at the late mature stage and minimal at the early mature and overmature stages.

Previous studies have investigated the relationship between extractable OM content and shale porosity for marine shale,^68^ while few studies have explored their correlation for transitional shale due to the fact that type III kerogen tends to generate relatively low amounts of liquid hydrocarbons. Xi et al.^65^ pointed out that there were no significant changes in pore volume and pore size distribution after extraction for transitional shale in northern China, and the decreases in mesopore and macropore volumes were probably related to compaction. In this study, the influence of free hydrocarbons (S_1_) and residual oil on pore volume was investigated. S_1_ represents the free hydrocarbons measured by Rock-Eval pyrolysis. Residual oil is tested by extracting the soluble OM using a Soxhlet instrument. Essentially, they have the same meaning, though they are measured by different methods. The correlations of S_1_ and residual oil with the volumes of micropores, mesopores, and macropores for the original sample and simulated samples are plotted in Figure 12. Micropore volume, mesopore volume, and macropore volume all exhibit negative correlations with the S_1_ value, with R^2^ values of 0.74, 0.58, and 0.37, respectively (Figure 12,a,c,e). Similarly, residual oil also displays significantly negative relationships with micropore volume, mesopore volume, and macropore volume, with R^2^ values of 0.81, 0.70, and 0.69, respectively (Figure 12,a,d,f). This suggests that extractable OMs fill and block shale pores in the studied original and simulated samples. Additionally, it can be concluded that extractable OM has the strongest influence on micropores, followed by mesopores, and lastly macropores. Furthermore, mesopores and macropores are also influenced by compaction, resulting in the R^2^ values for mesopores and macropores being lower than those for micropores.

**Figure 12:**
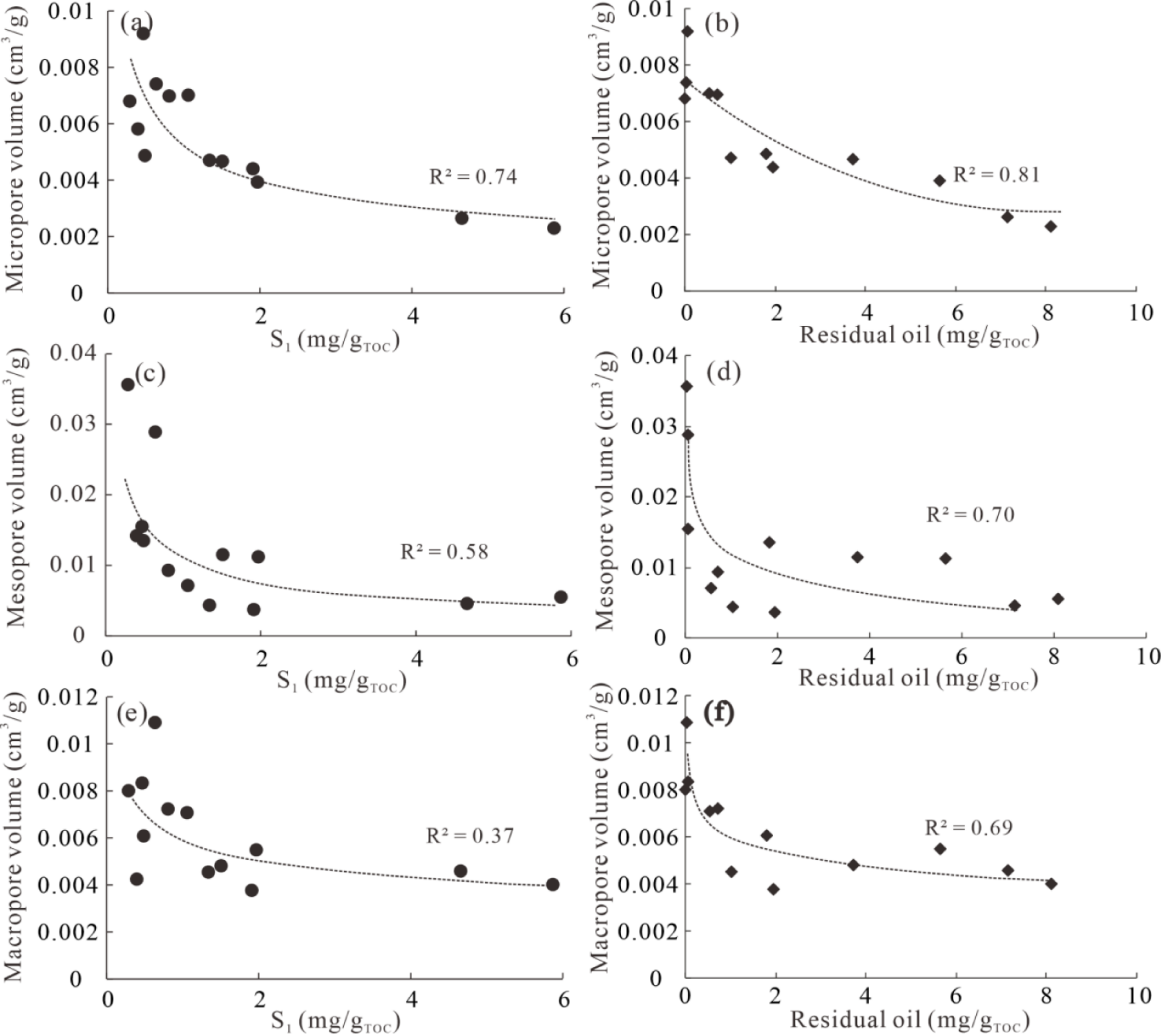
Crossplots of S_1_ and residual oil with volumes of micropores (a-b), mesopores (c-d), and macropores (e-f).

### 4.3 Response of OM Pore Evolution to the TOC Content

Many studies have shown that TOC content controls the total porosity to some extent in marine shale reservoirs,^69^ and shale with higher TOC content has a higher porosity and a larger surface area.^70^ The development of OM pores is a function of the decomposition of OM. As reported, the porosity of shale can increase by 4.9% when 35% of the OM is decomposed.^1^ However, no clear relationships could be observed between TOC content and pore volume in transitional shale in northern China.^65^ Cao et al.^39^ also observed no relationships among surface area, pore volume, and TOC content for the samples of the Longtan Formation with type III kerogen, even though the thermal maturities have a wide range in the Sichuan Basin. This suggests that OM pores are not well-developed in the gas-prone shale reservoir.^71^ However, the TOC content in these studies is static, and OM has undergone a hydrocarbon generation process, which cannot reflect the OM pore variation from the initial state to the present state in OM grains.

In this study, the influence of the TOC conversion rate on the shale pore volume was discussed. It can be seen that there is a positive relationship between the TOC conversion rate and micropore volume (Figure 13), with a correlation coefficient R^2^ = 0.55, suggesting that micropore volume increases with the decomposition of OM. Mesopore volume first displays a decreasing trend and subsequently an increasing trend with the TOC conversion rate (Figure 13), because extractable OM initially fills mesopores and reduces mesopore volume, and subsequently, extractable OM cracks into hydrocarbon gas, leading to the combination of micropores into mesopores. Therefore, a weakly positive correlation exists between the TOC conversion rate and mesopore volume, with R^2^ = 0.38. Macropore volume is also linearly correlated with the TOC conversion rate overall (Figure 13), with R^2^ = 0.66, due to the continued production of OM pores from solid bitumen and even vitrinite grains, as well as the limited influence of extractable OMs on macropores . Dominated by mesopore volume, the correlation between total pore volume and the TOC conversion rate exhibits a similar trend to mesopore volume (Figure 13), but it has a better correlation due to the combined influence of micropores and mesopores. Therefore, the low-TOC sample has higher pore volume and surface area, as shown in Figure 13 and Table 5 is due to the higher thermal maturity. In other words, the formation of OM pores is due to the conversion and decomposition of OM as a function of increased thermal maturity.

**Figure 13:**
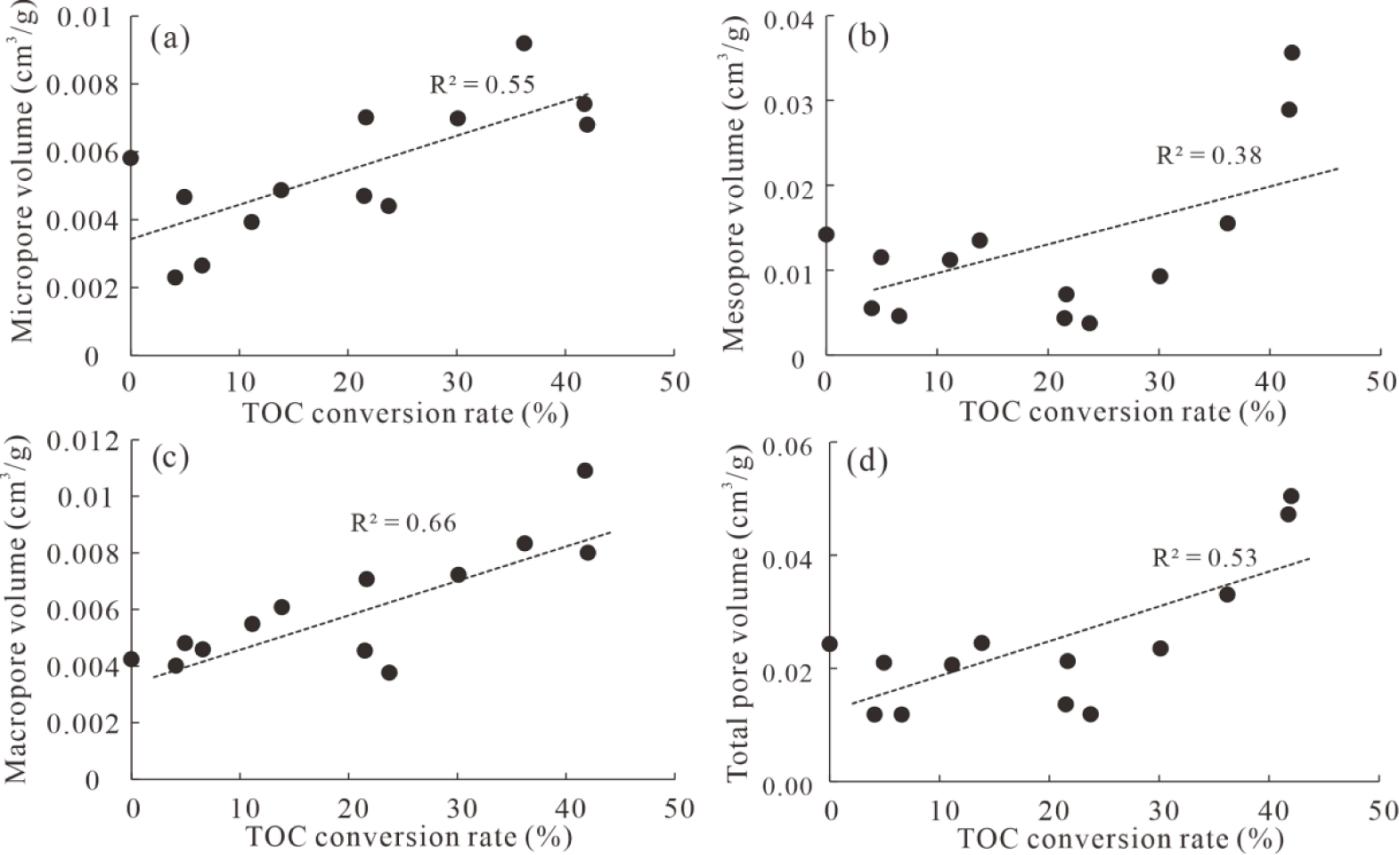
Crossplots of TOC conversion with volume of micropores (a), mesopores (b), macropores (c), and total pores (d).

### 4.4 Response of OM Pore Evolution to Thermal Maturation

Thermal maturity has an essential effect on shale pore development. Increasing thermal maturity can result in OM transformation, hydrocarbon generation, retention, expulsion and diverse types of pore formation.^72^ OM porosity increases with increasing thermal maturity and subsequently decreases at the overmature stage, as observed in many studies.^73^ However, investigations of thermal maturity in previous studies on OM pore development were mainly focused on simulated samples of marine facies and naturally evolved transitional shale samples.^39,74^ The dynamic evolution of OM pores and its relationship with thermal maturity in transitional shale across a series of maturity levels are still less studied.

To further explain the evolution of shale pores during this process, variations in the volumes of micropores, mesopores, macropores, and total pores with increased thermal maturity are plotted in Figure 14. As shown in Figure 14, micropore volume first displays a rapid decreasing trend at an Ro value of 0.47–1.02%, with the lowest value at an Ro value of 1.02%, due to the infilling of extractable OM at this stage. When the Ro value increases from 1.02% to 2.99%, micropore volume increases rapidly due to the decomposition of OM and the formation of porous solid bitumen. As Ro increases to 3.31–3.82%, micropore volume begins to decrease, probably due to the combination of micropores into mesopores and the graphitization of OM.^65^ Compared with micropores, mesopore volume displays different trends with an increased Ro value, as shown in Figure 14. Mesopore volume shows a rapid decrease at an Ro value of 0.47–1.56%, with the lowest value at Ro = 1.56%, and subsequently a rapid increase until the end of the simulation experiments. The former is also influenced by the infilling of extractable OM, while the latter is due to the cracking of extractable OM and the combination of micropores. The variation trend of macropore volume with increasing Ro value is similar to that of the micropore (Figure 14). However, the degree of influence of thermal maturity on macropore volume is significantly weaker, as the decomposition of OM generally generates extractable OM, which then fills micropores rather than macropores. The decrease in macropore volume at Ro = 3.31–3.82% is probably due to strong compaction. The variation trend of total pore volume with increasing Ro value is similar to that of mesopore volume (Figure 14), due to the fact that total pore volume is dominated by mesopores.

**Figure 14:**
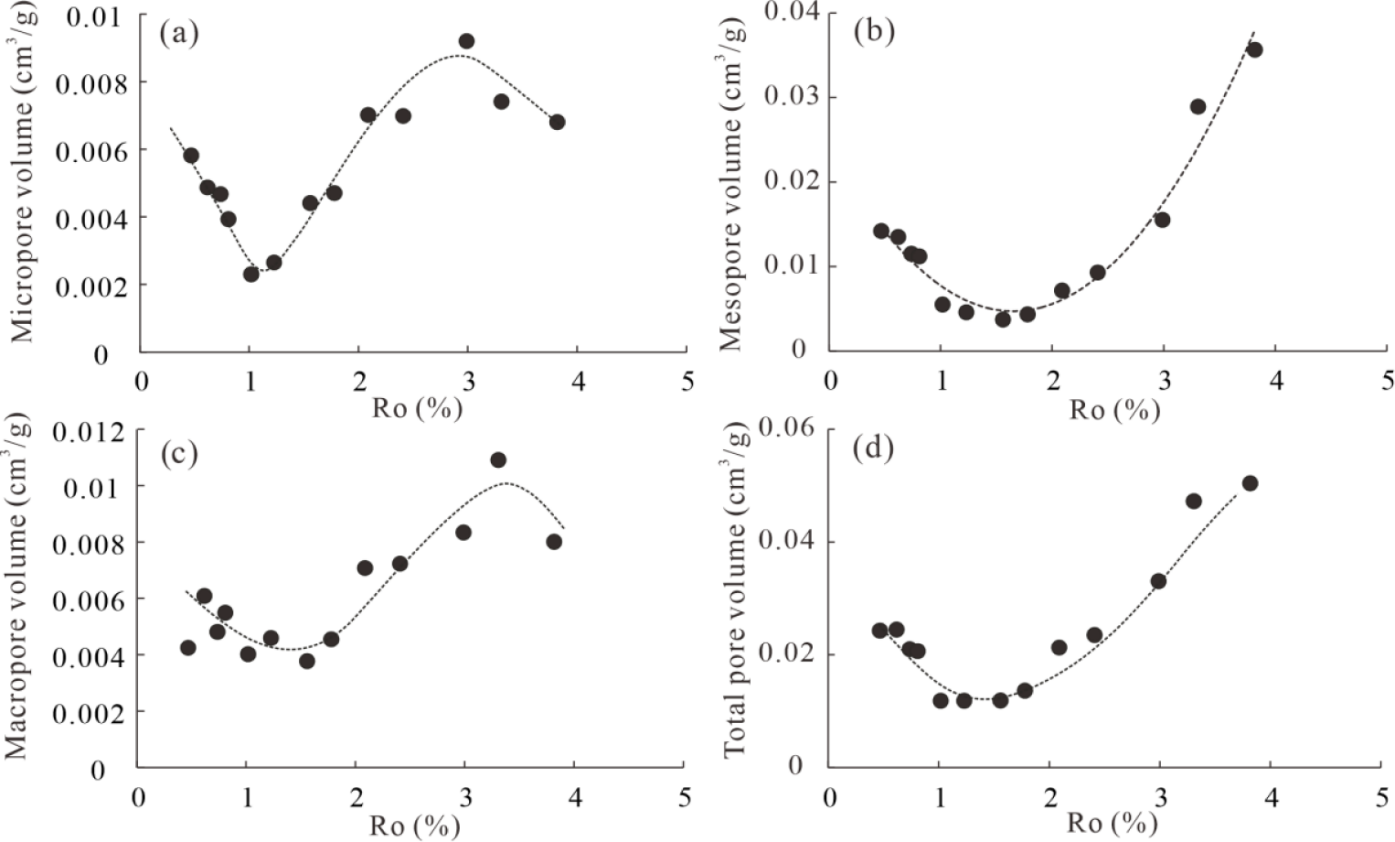
Crossplots of Ro value with volume of micropores (a), mesopores (b), macropores (c), and total pores (d).

### 4.5 Pore Evolution Stage in Transitional Shale

During the hydrocarbon generation process, significant changes in pore structure characteristics occur in the maturation sequence from the immature sample PLG (Ro = 0.47%) to the overmature sample PLG-600 at the dry gas window (Ro = 3.82%). Pore structure heterogeneity varies dramatically due to the transformation of different types of pores and the retention or expulsion of oil and gas.

According to the results of this study, the pore evolution stage can be summarized in Figure 15. At the immature stage (Ro < 0.5%), the original sample PLG has a larger micropore volume, mesopore volume, and specific surface area, which indicates that thermally immature transitional shale may host many primary pores.^65^ In fact, a certain amount of primary pores are observed in the FE-SEM images (Figure 6), including a certain amount of OM pores, despite the fact that there are many nonporous immature OM particles. At the oil generation stage (0.5% < Ro < 1.1%), oil and gas start to generate, and most oil is retained in the shale. At this stage, the surface areas and volumes of micropores and mesopores decrease rapidly, and surface area and micropore volume reach their minimum values when the Ro value is 1.02%. Meanwhile, macropore volume decreases slowly at this stage. The decreases in micropore volume and surface area are primarily due to the fact that micropores have larger surface areas that are easily infilled by extractable OM generated via kerogen pyrolysis.^60^ The reduction of mesopore volume is controlled by extractable OM infilling and compaction, while the reduction of macropore volume is mainly controlled by compaction.^62^ At postoil generation stage (1.1% < Ro < 1.5%), the residual oil reaches the maximum value at Ro = 1.23%. During this period, micropore volume and surface area show an increasing trend due to the cracking of extractable OM and the release of hydrocarbon,^35^ which form nearly circular pores with smaller diameters in the OM grains (Figure 7,h). The decrease in mesopore volume is also due to the infilling of extractable OM and compaction. The change in macropore volume is not obvious, due to the balance between the increase in pores from hydrocarbon generation and the reduction in pores from compaction. At the wet gas generation stage (1.5% < Ro < 2%), the slight increase in volumes and surface areas of micropores, mesopores, and macropores might result from a decrease in residual oil and an increase in expelled oil. Meanwhile, macropores are gradually formed by the connection of micropores and mesopores, which is consistent with previous studies of natural shale.^65,75^ At dry gas generation stage (Ro > 2%), surface areas and the volumes of micropore and macropore first increase and then decrease and mesopore always shows an increasing trend. Although compaction still has impact on pore development, the loss of pore volume is determined by the sizes of pores. During this period, the porous solid bitumen formation and condensation from oil evolution can provide a large number of micropores and mesopores, and the newly formed micropores and mesopores are easily to be preserved because the small pore diameter increases their collapse strength that shields them from compaction, and stronger molecular forces also help withstand high pressures.^76^ The transformation and destruction of pores occur with further increased thermal maturation. On one hand, highly overmature shale results in organic carbonization, leading to a decrease in micropore volume and an increase in mesopore volume due to the combination of micropores. On the other hand, decreased macropore volume may be caused by postcompaction.

**Figure 15:**
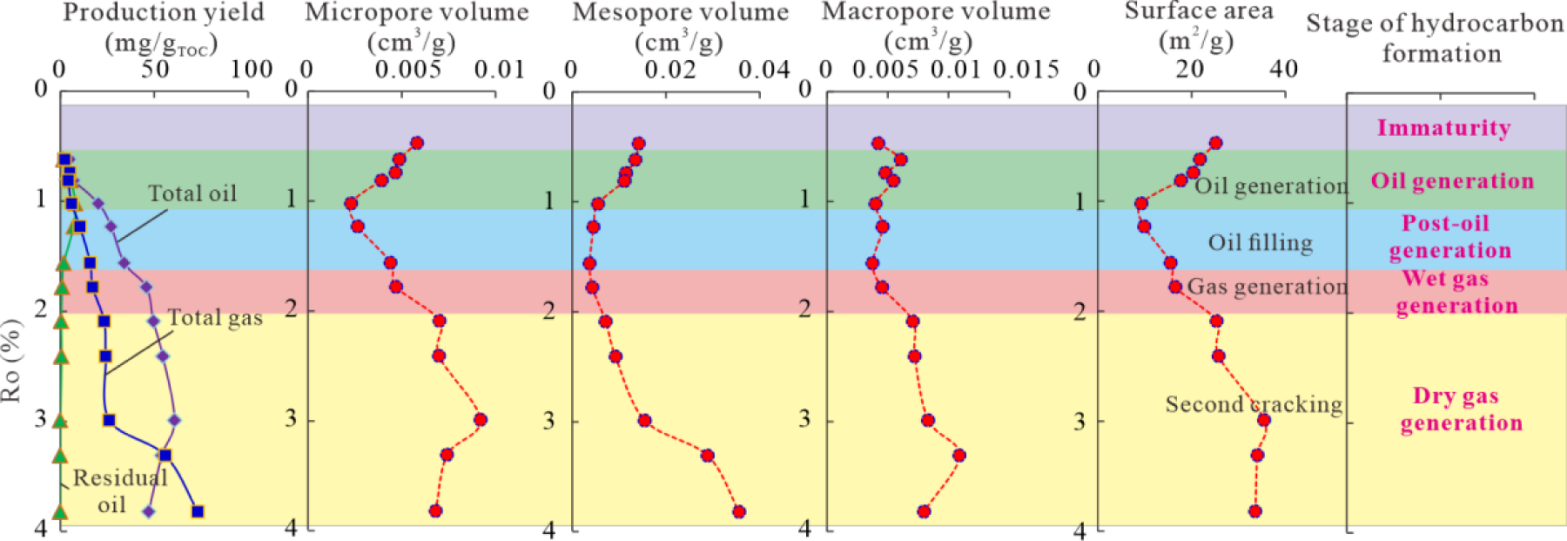
Pore evolution stage accompanied by hydrocarbon generation and expulsion during thermal maturation.

### 4.6 OM Porosity Evolution Model

To visually illustrate the development and evolution of OM pores in transitional shale, a pore evolution model spanning from immaturity to over-maturity was established and compared with that of marine shale (Figure 16). The development of OM pores in marine shale experiences four stages.^54^ In the early oil window, the generated oil, in the form of remobilized solid bitumen, fills nearby pores, with few pores being visible in the oil-prone kerogen. The pore volume exhibits a significant decreasing trend due to the retained oil filling OM pores, fractures, and interparticle pores, forming a connected solid bitumen network in the shale. With increasing thermal maturation, a large number of OM spongy pores are generated as residual oil cracks into hydrocarbon gas. This process releases the blocked pores and creates new OM pores, which significantly increase OM porosity. The pore volume reaches its maximum value at Ro values of 2.5–2.9%.^39^ However, with further increases in thermal maturation, a significant reduction in pore volume occurs due to strong compaction.

**Figure 16:**
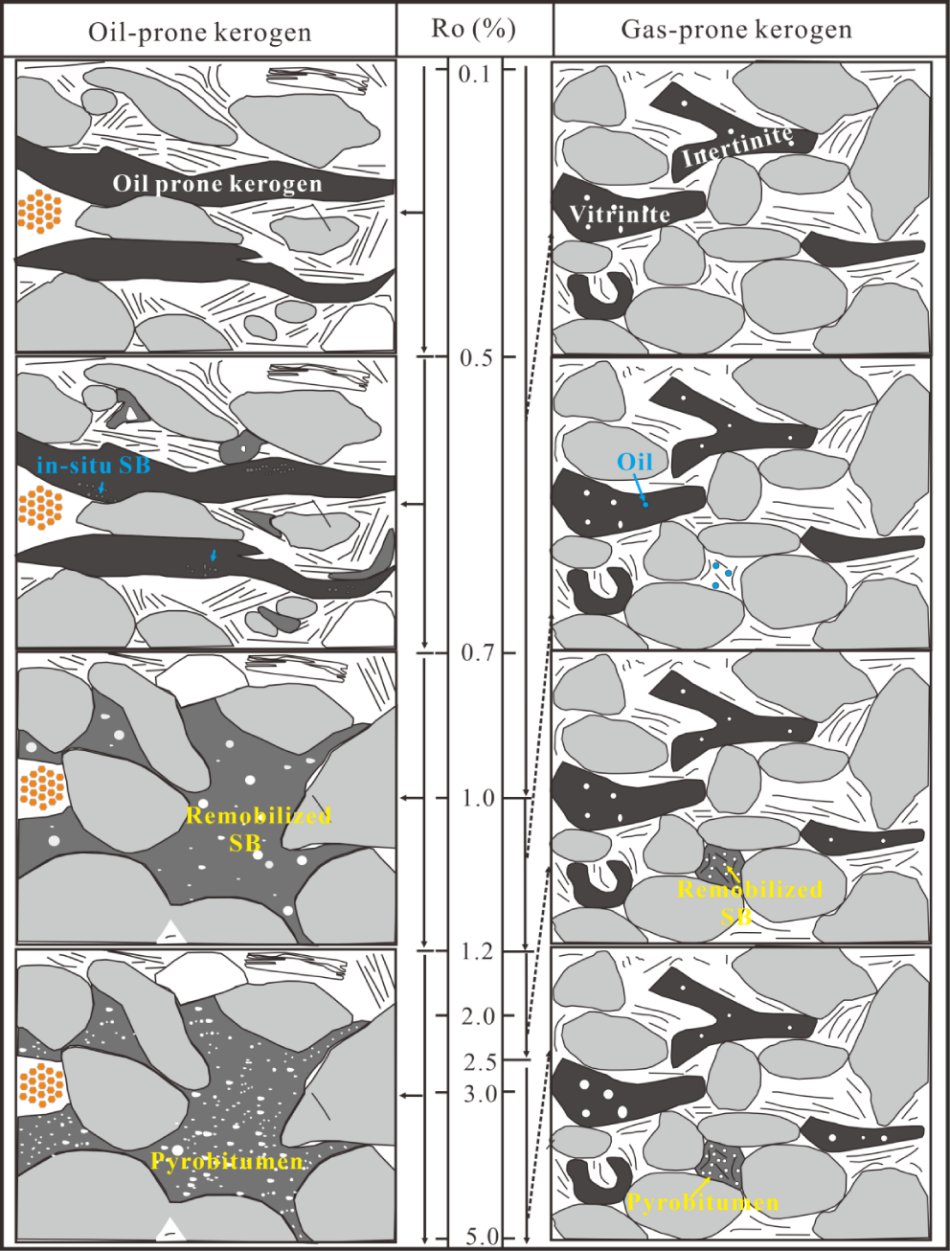
OM pore evolution models from immature to overmature stages for oil-prone and gas-prone kerogen.

As a comparison, liquid hydrocarbon and associated OM pores are poorly developed in transitional shale dominated by type II_2_–III kerogen.^77^ Vitrinite and inertinite particles generally generate few pores through the thermal evolution process due to their low hydrocarbon generation capacity. The aliphatic functional groups in vitrinite have been oxidized to some extent during the migration and deposition process.^78^ It can also be observed that inertinite and vitrinite grains do not display a noticeable change in morphology throughout the entire maturity range (Figure 16). Additionally, only several cellular pores are developed in inertinite and vitrinite grains, with diameters ranging from several tens to hundreds of nanometers. From another perspective, this type of kerogen can resist compaction, protect internal pores, and maintain its original shape during burial. In the oil window stage, a certain amount of oil is generated and migrates to nearby OM pores, which significantly reduces the shale pore volume. With increasing thermal maturation, oil with high mobility is converted into gas, and a substantial number of OM pores can be developed. After entering the overmature stage, more OM pores are developed within pyrobitumen, which can significantly increase OM micropores and mesopores. Meanwhile, OM pores in vitrinite are also enlarged due to continued hydrocarbon gas generation. OM pore development in type III kerogen occurs mainly via the removal of functional groups with low to high bond energies,^40^ resulting in the continuous formation of OM pores in vitrinite before OM graphitization,^64^ though the number of OM pores remains limited .

## 5 Conclusions

The OM pore development characteristics and evolution process of transitional shale during the entire hydrocarbon generation process were investigated via thermal simulation experiments on immature Shanxi Formation shale, and the following conclusions have been drawn.

(1) In terms of semi-closed pyrolysis experiments, the maximum yields of oil and gas from the Shanxi Formation shale are 60.95 mg/g_TOC_ and 412.50 mL/g_TOC_, respectively, at a heating rate of 2 °C/h. The highest total oil production occurs at 500 °C, while total gas production continues to increase until the end of the pyrolysis experiments. The transitional shale of the Shanxi Formation also has the ability to generate oil, with most of it being expelled from the shale reservoir.

(2) The original shale with immaturity contains some unique cellular pores in vitrinite and inertinite grains. The morphologies of kerogen grains and OM pores are not greatly affected by thermal maturation. During pyrolysis experiments, OM pores can be clearly observed at temperatures of 275–300 °C, and subsequently, some disappear at 320–385 °C. Finally, OM pores are enlarged, and newly formed OM pores are observed after 425 °C.

(3) OM pore development in transitional shale is significantly controlled by the hydrocarbon generation process. Micropore volume first shows a rapid decrease with increasing hydrocarbon generation, reaching its lowest value at Ro = 1.02%, followed by a rapid increase due to newly formed pores in kerogen associated with abundant hydrocarbon gas generation, and finally a slight decrease at extremely high thermal maturation due to the depletion of the generation capacity of kerogen. Mesopore volume initially experiences a rapid decreasing trend due to extractable OM infilling, followed by a rapid increasing trend due to the decomposition of oil and kerogen and the combination of micropores. Macropore volume variation is similar to micropore volume, but the decreasing trend at the extremely high thermal maturation stage is attributed to strong compaction.

(4) Accordingly, OM pore evolution can be divided into four stages, and a model of OM pore evolution is also established. At the oil generation stage (0.5% < Ro < 1.1%), residual oil can fill OM pores and significantly reduce shale pore volume. At the post-oil generation stage (1.1% < Ro < 1.5%), extractable OM migrates and fills mesopores, and micropore volume increases due to the partial cracking of extractable OM and hydrocarbon gas produced from kerogen. At the wet gas generation stage (1.5% < Ro < 2%), shale pore volume increases due to the release of occupied pores. At the dry gas generation stage (Ro > 2%), shale pore volume increases rapidly when Ro is below 2.99%, due to the formation of OM pores in pyrobitumen and newly formed pores in kerogen. When Ro is above 3.31%, the graphitization of OM and compaction can reduce micropore volume and macropore volume, respectively.
